# Multi-Family Therapy for First Episode Psychosis: Experiences of Families in Singapore

**DOI:** 10.3389/fpsyt.2021.788827

**Published:** 2021-12-24

**Authors:** Christopher Loh, Wilfred Liang, Helen Lee, Astelle Koh

**Affiliations:** ^1^Adult Eating Disorders Service, South West London and St. George's National Health Service (NHS) Trust, London, United Kingdom; ^2^Early Psychosis Intervention Programme, Institute of Mental Health, Singapore, Singapore; ^3^Medical Social Services, Ng Teng Fong General Hospital and Jurong Community Hospital, National University Health System, Singapore, Singapore

**Keywords:** psychosis, first episode psychosis, multi-family therapy (MFT), multiple family group therapy, multiple family group programme, early intervention in psychosis, culturally sensitive adaptation

## Abstract

**Aim:** This qualitative study examined the experiences of families with Multi-Family Therapy (MFT) provided by the Early Psychosis Intervention Programme (EPIP) in Singapore. The MFT was piloted over a period of 2 years and findings from this study were used to further refine the MFT to better meet the needs of Singaporean families in the service.

**Methods:** Families who completed the MFT were invited to participate in the study. Nine clients and ten carers who consented to participate in the study were allocated to two client and two carer Focus Group Discussions (FGDs) respectively. A semi-structured interview schedule was used to facilitate the discussions. The FGDs were audio recorded, transcribed, and anonymised. The data was analysed using thematic analysis.

**Results:** Four main themes emerged from the analysis: (1) therapeutic processes of MFT, (2) positive changes in family relationships, (3) improvements in coping with psychosis, and (4) suggestions for improvement in MFT. The families suggested some structural changes to the MFT, and more carers than clients would prefer therapists to offer more expert advice.

**Conclusions:** Findings suggest that a Western-based MFT can be adapted to work with Singaporean families. This study sheds light on the therapeutic processes that may be related to the changes in family relationships and coping with psychosis. In addition, it suggests that therapists taking an expert and authoritative approach may not fit with the needs of younger generations in Singapore. It advocates for therapists to take a flexible and fluid stance to work with Singaporean families.

## Introduction

Multi-Family Therapy (MFT) is a Western-based model that combines the theory and practise of group and family therapies, and its key therapeutic aim is to foster mutual support and learning between families who face similar difficulties (1). Over the last seven decades, MFT has been applied across the world in various settings such as psychosis (2–6), mood disorders (7), eating disorders (8), child and adolescent mental health (9–11), family separation and divorce (12), physical health and educational settings (13). The wide range of MFT models and settings makes it difficult to establish a solid evidence base. A narrative review suggested that MFT has the strongest evidence base for psychosis in comparison to other settings (14). By and large, MFT in psychosis was based on the earlier psychoeducation model developed by McFarlane (15). Over the years, MFT in the field of psychosis has evolved into two different strands: one that preserves a strong psychoeducational component (16, 17), and the other that focuses on therapeutic activities (1, 18). Most adaptations of MFT to work with Asian families draw on a psychoeducational model (3–6) and it was only recently that a MFT based on therapeutic activities was developed in Singapore by same group of authors in this current study (19).

More broadly, family-based interventions—albeit different in their theoretical approach, method, and techniques—share similar aims to support engagement with service, treatment adherence, symptoms reduction, relapse prevention, and improvement in carer's well-being (20, 21). Previous MFT studies found that this particular family-based intervention helped to improve the patients' quality of life and carers' knowledge of illness (6), reduce carer burden (4, 5), reduce hospitalisation rate, and support better engagement with mental health services (3). Although the benefits of MFT are well-documented in the field of psychosis, a systematic review highlighted the wide range of variations in this particular therapeutic intervention (22). For instance, Kung et al. (6) piloted a MFT specifically for Chinese-American patients with schizophrenia. This model adopted a psychoeducational and problem-solving approach using a combination of multi-family groups for carers only (without the clients) and single family therapy sessions. Chien and Wong (5) described a MFT which comprised 18 2-h sessions that were conducted on a fortnightly basis over four key stages: (1) three sessions of orientation and engagement, (2) six sessions of educational workshop, (3) seven sessions that focussed on therapeutic family role and strength building and (4) two sessions on termination. Whilst different forms of MFT to work with Asian families continue to develop in the field of psychosis, it is imperative for therapists to be sensitive to the cultural nuances when applying a Western model to the local context (23–25). Although incompatibilities exist between Western-based treatment models and Asian cultures, Bentelspacher et al. (26) indicated that multi-family psychoeducational groups could be beneficial to Singaporean families when adjustments were made to the process of facilitation to suit the local cultural norms and values. Chinese Singaporean clients' aetiology and treatment beliefs about psychological problems are eclectic, deriving from both Western and traditional therapies (27). Therefore, counselling and psychotherapy approaches combining both Western and traditional therapies would more likely fit with the needs of Chinese Singaporeans (28).

Prior to this study, the MFT undergone a 2-year pilot phase to establish the structure and content of the programme as well as understand the facilitation process that would best meet the needs of Singaporean families. Preliminary results from the pilot study were promising (19). This MFT was an activity-based programme grounded in psychotherapeutic principles which comprised 4 weekly sessions and a follow up session. The therapeutic activities included “Image of Psychosis,” “Multi-family Group Discussion,” “Family Tree,” “Sculpting,” “Letter to Psychosis,” “Family Life River,” and “Self-Psychoeducation and Consolidation of Work” [see (19) for further details of the MFT]. The objectives of the MFT were: (1) to improve understanding of psychosis and its impact on clients and their families, (2) to provide a space for families to learn from one another about new ways of managing psychosis, (3) to provide opportunities for families with similar experiences to expand their support network, and (4) to help families draw on their resources to manage psychosis. This qualitative study aimed to explore the experiences of Singaporean families with MFT in EPIP. The position of this study was influenced by several concerns: (a) there is a need to understand more about the processes rather than outcomes of MFT, (b) it is crucial to look into the finer details of adapting a Western-based model to a specific localised context (in this case Singapore) and (c) there is an interest to “research with” rather than “research on” the families who were coping with psychosis. The study served to generate rather than test hypotheses. The hypotheses generated may be tested in future research to further understand the use of MFT in Singapore. In addition, findings from the study will be used for service evaluation and development.

## Methods

The study was approved by the National Healthcare Group (NHG) Domain Specific Review Board (DSRB) and the Institute of Mental Health (IMH) Institutional Research Review Committee (IRRC) in Singapore. A total of 16 families took part in a course of the MFT and three families dropped out which gave an 81.25% of completion rate.

### Participants

This single site study utilised a purposeful sampling method whereby participants were recruited *via* the MFT in EPIP over a period of a year. A researcher introduced the study in the first MFT session and gave a follow up call to the clients and carers after 2 weeks to ask if they would like to take part in the study. Written informed consent was obtained from clients and carers who consented to take part in the study. The inclusion criteria for clients were: (1) EPIP client who presented with first episode psychosis, (2) between the age of 16 and 40, (3) Singaporean or Singapore Permanent Resident, (4) clinically stable, (5) English speaking and (6) completed a course of MFT. The inclusion criteria for carers were: (i) family member of an EPIP client, (ii) aged 21 and above, (iii) Singaporean or Singapore Permanent Resident, (iv) English speaking and (v) completed a course of MFT. The exclusion criteria for both clients and carers were: (a) client/carer who was not fluent in English and (b) not willing for the interview to be audio recorded. For younger EPIP clients aged 16 to 20, parental consent was obtained.

A total of nine clients and 10 carers participated in a Focus Group Discussion (FGD). The participants' demographics are summarised in Table 1. The proportion of participants' ethnicity was representative of Singapore (and EPIP) population which consists of 74.2% Chinese, 13.7% Malays, 8.9% Indians and 3.2 other ethnicities (29). All clients had a family member (i.e., their carer) who took part in this study. Only one carer did not have a family member (i.e., the client) participating together with them as the client declined the invitation. Each participant was given a small monetary remuneration upon completing the FGD.

**Table 1:** Participants' demographics.

	***N***	**%**
**Gender**		
Male	11	57.9
Female	8	42.1
**Ethnicity**		
Chinese	15	78.9
Malay	4	21.1
**Age of the clients (mean, SD)**	(21.1, 2.85)	
**Relationship of carer with the clients**		
Mother	6	60
Father	3	30
Brother	1	10

### Data Collection

The FGDs were conducted by a facilitator with a note taker who were part of the EPIP team and not blinded to the study. The FGDs took place immediately after the final session of MFT. The discussions were facilitated using a semi-structured interview schedule (see Appendix 1). Four FGDs were organised for two different groups of participants: one for clients and the other for carers. The clients and carers were grouped separately with the purpose to facilitate more open discussion. Each FGD lasted ~1 h and comprised 4–5 participants. The FGDs were audio recorded and transcribed to anonymised verbatim reports, and the researchers checked the transcripts for consistency.

### Data Analysis

The data was analysed using thematic analysis which involved identifying, analysing, and reporting patterns (themes) (30). The analysis was conducted manually by using an excel sheet to label, organise and sort the codes and themes accordingly. Data analysis commenced with two researchers familiarising themselves by reading and re-reading of the transcripts and generating a list of ideas for coding. Next, the raw data was organised into units of analysis and each unit was summarised to create a list of initial codes. The list of initial codes was then organised into meaningful groups (themes). Due to the exploratory nature of the study, coding of the data was done using a data-driven approach (31) where two researchers approached the data with no specific questions in mind to allow themes to emerge. A codebook was developed and applied to a sub-sample (20%) of the data to verify its applicability by the two researchers. Throughout this process, a third researcher was involved when there were discrepancies in the coding process. The three researchers considered how the overarching themes were related to one another and how they fitted with the overall purpose of the study. When the analysis was satisfactory, a thematic map was produced to reflect the meaning of the data as a whole.

## Results

The emerging themes from the FGDs were categorised into four overarching themes: (1) therapeutic processes of MFT, (2) positive changes in family relationships, (3) improvements in coping with psychosis and (4) suggestions for improvement in MFT. These overarching themes were found in both clients' and carers' groups which reflected the similar experiences shared between both clients and carers.

### Therapeutic Processes of MFT

This theme sheds light on the mechanisms of MFT. Participants felt validated and comforted by others who were in similar situation; they felt that they were not alone. They reported feeling safe which helped them to share openly with each other.

*Carer08:* “*I think it's basically a programme which provides us with a great opportunity to actually meet with fellow parents who have got children going through this condition itself, otherwise you would feel kind of alone*.”

*Carer04:* “*From their stories similar to my daughter, and I know that I am not alone here, and there is somebody with me*.”

*Client02:* “*Basically what I feel is it is a place where we do not judge each other, help our relationships to be better... and basically help us to enable... be a bit more frank about things such [as] psychosis…*”

*Client06:* “*Maybe more... more people can tell more about their condition without hiding*.”

Some participants reported that MFT created the opportunity for them to connect with others and expand their support network, which they were keen to maintain beyond the sessions.

*Client03:* “*I see this as a way to build a community of people who have similar experiences …*”

*Carer05:* “*But later on we meet up again, and we have a common call. So, this actually helped in a progressive manner*.”

Participants also felt that there was mutual learning amongst themselves.

*Client06:* “*Because there is also other families, so we can learn from each other even though different people have different type of illness*.”

*Client04:* “*We shared a lot of experience and learn from each other. But I think by talking about the issues, I think we have more like self-aware[ness], like [knowing] what's going on you know*.”

### Positive Changes in Family Relationship

Participants reported that there was more empathy and understanding towards each other within the family unit.

*Client09:* “*For me I think it would be good if our family knows that we are getting improve in our sickness. So they understand us well*.”

*Carer02:* “…* like as a family, it really helps us to realign our expectations …*”

Some participants noticed that there was more communication and positive interactions with their family members. It encouraged them to have conversations which they would not otherwise have in the context of home. Some of them also reported increased family cohesion and unity.

*Carer05:* “*First it gives me the opportunity to present my thoughts to my son. On one to one basis we normally don't talk about this but in this set up itself, the presentation itself give me opportunity to say a bit more about myself, what I am thinking of to my son*.”

*Carer05:* “*We change our habit and we are more united on what we see*.”

*Carer03:* “*I think more closer now especially to the dad …*”

### Improvements in Coping With Psychosis

Besides the interpersonal gains as mentioned above, there were accounts of intrapersonal gains in terms of improving one's capacity in dealing with psychosis. Participants reported gaining a better understanding of and coming to terms with the illness.

*Carer07:* “…* he is also frustrated why he has this illness. So, after going through all these, slowly he understands more of it*.”

*Client07:* “*It's more like ya just understanding of the illness itself, and kind of explaining your own symptoms to your own parents… not just to other families*.”

Participants also reported acquiring new skills to cope with psychosis on a day-to-day basis through MFT.

*Client07:* “…* it kind of brought the awareness to me… like I am aware of this now so how can I like… start to cope with it*.”

*Client08:* “*It would help us in our everyday … it will help us to learn how to cope better with our everyday tasks with our condition..*.”

### Suggestions for Improvements

There were constructive suggestions to make some structural changes to the programme.

*Client01:* “*I think there should be some breakouts… more breakouts I would say, because certain things, you would see that in front of the loved ones, I think caregivers are also holding back certain things*.”

*Client08:* “*I think maybe they can let us do some exercises together like as a family or like [in] pair[s] to learn more about other people also*.”

Participants who were mostly carers also expressed their preference for more professional advice during MFT.

*Carer02:* “*I feel that certain topics need that expertise to just tell us at the end are we on the right track and is this the right way*.”

*Carer03:* “*What are people in the field talking about it? Like what is this illness about? I would like to know more reliable information*.”

## Discussion

The current article explores the experiences of families with MFT in the context of EPIP in Singapore. Findings from this study provide insights to the processes of MFT that may account for the changes in the families. Constructive feedback from participants may be of direct clinical relevance for service development to better suit the needs of Singaporean families.

### Processes of MFT

A safe environment is the foundation for therapeutic work to take place in any forms of therapy. Clients and carers in the current study felt that they were not alone in the situation. This reflects the connection between the group participants and the sense of safety in the group that was established over the course of MFT. The process of creating a safe space in MFT is a joint effort between participants and therapists as illustrated in a study (11). The essence of MFT is to draw on the resources in the group whereby therapists “de-center”—that is becoming less active—over time and enable participants to take more lead in the work (1). In working with EPIP clients and their families, the notion of therapists de-centering looked different in that therapists still took a fairly active role, alongside holding an expert and authoritative stance, throughout the course of MFT.

It is well-documented that mutual support and learning are key therapeutic factors of MFT in working with Asian families (6, 10, 26). Reports from clients and carers in the current study echo findings from these Asian studies. Mutual learning and support were only plausible when families participated openly in the group discussions. Both clients' and carers' accounts of mutual support and learning were particularly pertinent to working with Singaporean families as it challenged the view that they were unlikely to engage in such a group setting due to belief about “not washing your dirty linen in public.” Furthermore, the clients' and carers' engagement throughout the MFT also challenged the view that Singaporean families were unlikely to seek help due to stigma of mental illness. The safe space that was co-created between participants and therapists warmed the context for open sharing and communication, which in turn may have facilitated support and learning in the group. As participants felt heard and validated, it may have positioned them to share more openly and, thereby, further fortified the sense of safety in the group.

Another key finding is the shared experience between clients and carers with the positive changes in familial relationships where there was more empathy, communication, and understanding. The safe therapeutic space could help families to have conversations that were otherwise difficult to have at home. Besides providing psychoeducation, MFT has the potential to strengthen relationships. It could also prevent the risk of relationship breakdown due to psychosis. Several studies in Asia has suggested on the use of MFT to enhance familial relationships (9–12). More importantly, there was a greater sense of family cohesion reported by clients and carers. The processes outlined above may be related to the shifts in the familial communication and relationships where there was, perhaps, a sense of esprit de corps in fighting psychosis as a family unit. Changes in relationship may also be linked to reduced carer burden as reported in some MFT studies in a psychosis setting in Hong Kong (4, 5); clients and their families carers are likely in a better place to work on their relationship when there is reduced carer burden. Expressed Emotion (EE) is associated with family burden in families of clients with first episode psychosis (32) and there is evidence to support the use of family-based interventions in reducing EE (33). Besides addressing carer burden, MFT may have the potential to alleviate EE in familial relationships. However, there is no universal norm in EE experience and thus it is important to be aware that care provision and care receiving behaviours differ across cultures (34).

In general, there was an improvement in subjective experience of coping with psychosis. Consistent with previous studies (6, 9), participants reported that MFT helped them to understand more about the illness. In addition, they acquired new cope skills and found themselves coming more to terms with psychosis. With increased confidence in coping and more acceptance of the illness, clients and carers may be more enabled to address the relational impact of psychosis.

### Ideas for Service Development

Reports of clients and carers revealed some differences in the way the two groups preferred the MFT to be conducted. Clients would like to have more multi-family group activities to bond and have discussions with their families. Carers, on the contrary, expressed the need to have more discussions amongst themselves without the clients as they felt a need to hold back. A benefit of MFT is the ability to offer both multi-family group activities and separate group discussions that cater to the two reported preferences. MFT can served as a stepping stone for clients and carers to be signposted to other services to suit their needs. Clients and their families, for instance, can be referred for single family therapy after completion of MFT. The need for carers to have more discussions with other carers can be fulfilled *via* attending carers' support groups in the community. Furthermore, carers (and clients) can be encouraged to form their own support network beyond the MFT by keeping in touch on messenger group chat and social media. It is worthwhile to note that some MFT groups managed to do so and found such support network helpful.

In line with previous studies which advocated for taking an expert and authoritative stance in working with Asian families (19, 24, 26, 35, 36), participants (mostly carers) indicated that they would like more professional input. A unique finding in the current study is that this feedback came from the carers rather than the clients. It is worth to reiterate here that the clients were young adults (with a mean age of 21.1) and the difference in preference for therapist's approach may be related to the difference in generation between young adult clients and their carers. This difference may be influenced by the changing traditional values and mindset over the generations in Singapore. As Foo et al. (27) highlighted, Chinese Singaporean clients' understanding of psychological issues and treatment are influenced by Western models and traditional local beliefs. Therefore, therapists need to be flexible in their approaches and it is crucial not to assume that taking an expert and authoritative stance would fit with Singaporean families.

### Self-Reflexivity

The four researchers are Singaporean Chinese in a life stage which was generally between that of the clients' and carers'. Two of them are men and two women. They were involved in the MFT in different ways. They were mindful of how their age, gender, ethnicity, and involvement in MFT may influence the way they understood and interpreted the participants' narratives. Thus, the authors met on many occasions to cross check with each other on their analyses with the aim to discuss any discrepancies as well as examine the influence of their potential biases on the study.

### Limitations

Most carers in this study were parents which meant that experiences of spouses/partners and siblings were not represented. The clients were mostly young adults and likely shared similar life stage concerns. Therefore, findings from this study may not be applicable to clients who are older in age. The MFT was piloted in English (the official language in Singapore) and most of the participants were Chinese Singaporeans. This could mean that MFT may not fit the needs of non-English speaking families in Singapore. Future MFT could recruit spouses/partners and siblings as well as clients who are older in age. It would be useful to include more families from other ethnic backgrounds and possibly conduct the MFT in other languages. Concomitantly, future MFT research could explore the experiences of these groups of families which were not represented in the current study. Finally, the therapists' experiences of the MFT are missing in this study. Perhaps future studies could also look into this area to understand the challenges of conducting the MFT and, more importantly, support the service provider in sustaining this therapeutic intervention.

## Conclusion

This project builds on the preliminary results from the pilot study on the use of MFT with EPIP clients and their families (19). Findings from this study support the argument that MFT can help families to cope better with psychosis and rebuild their relationships; they also endorse the view that a Western-based therapy model can be adapted to meet the needs of Singaporean families. What this study adds to the existing literature is that it highlights the therapeutic processes of MFT in helping Singaporean families in their recovery from psychosis. Interestingly, it also suggests that taking an expert and authoritative stance may not fit with the younger generations in Singapore as participants who expressed preference for this approach were carers but not young adult clients. Thus, the study calls for therapists to adopt a flexible and fluid stance that attends to different generations when working with Singaporean families.

## Data Availability Statement

The original contributions presented in the study are included in the article/supplementary material, further inquiries can be directed to the corresponding author.

## Ethics Statement

The studies involving human participants were reviewed and approved by National Healthcare Group (NHG) Domain Specific Review Board (DSRB) and Institute of Mental Health (IMH) Institutional Research Review Committee (IRRC). Written informed consent to participate in this study was provided by the participants' legal guardian/next of kin.

## Author Contributions

CL was the lead in the development of the MFT and this study. WL, HL, and AK supported the development of the MFT and all aspects of this study. All authors contributed to the article and approved the submitted version.

## Funding

This study was funded by the Centre Grant Research Seed Funding (Pilot Study) at the IMH. A total of SGD$2880 was provided.

## Conflict of Interest

WL is employed by Cigna Europe Insurance Company S.A.-N.V.,. The remaining authors declare that the research was conducted in the absence of any commercial or financial relationships that could be construed as a potential conflict of interest.

## Publisher's Note

All claims expressed in this article are solely those of the authors and do not necessarily represent those of their affiliated organizations, or those of the publisher, the editors and the reviewers. Any product that may be evaluated in this article, or claim that may be made by its manufacturer, is not guaranteed or endorsed by the publisher.

## Appendix 1

### MFT Focus Group Discussion

#### Interview Schedule

Main questions to answer in focus group discussion:

- What was the experience of young people/adults and their families with the MFT?
- How has the MFT impacted on the young people/adults and their families?
- What is a successful MFT to young people/adults and their families?

- *What was the experience of young people/adults and their families with the MFT?*

- What was your experience with the MFT Programme? [to elicit thoughts and feelings about the structure and content of the programme]
- Can you tell me about your experiences with the MFT Programme?
- Let's say you wanted to describe the programme to your friend, in your own words, how would you describe it?
- What do you think are some of the benefits of the programme? [Invite participants to list it out and discuss one benefit each. Slips can be collected later for text analysis]
- What would be some of your suggestions for improving the programme? [Invite participants to list it out and discuss one benefit each. Slips can be collected later for text analysis]
- Can you tell me what was the best part or experience in the programme?

- *How has the MFT impacted on the young people/adults and their families?*

- Have you noticed any effect the MFT has on you? [to elicit observations on the effects of the MFT and changes in selves]
- Do you think you have changed as a result of participating in the programme? Yes/No, can you tell me more about it?
- When you first joined the programme, what did you hope to get from the programme?
- Do you think the programme has met your needs? Yes/No, tell me more about it.
- Have you noticed any effect the MFT has on your family members who participated? [to elicit observations on the effects of the MFT and changes in family members].
- Do you think your family members have changed as a result of participating in the programme? Yes/No, can you tell me more about it?
- When your family members first joined the programme, what did they hope to get from the programme?
- Do you think the programme has met their needs? Yes/No, tell me more about it.

- *What is a successful MFT to young people/adults and their families?*

- How would you describe a MFT Programme that is successful? [to invite participants to define a successful programme]
- If your case manager said to you, the MFT Programme is a successful programme, what would it mean to you?
- How would you see a successful MFT Programme? What need to happen the MFT Programme?
- What would a successful MFT Programme do for you personally?
- What would a successful MFT Programme do for your family?
